# Within-Network Connectivity in the Salience Network After Attention Bias Modification Training in Residual Depression: Report From a Preregistered Clinical Trial

**DOI:** 10.3389/fnhum.2018.00508

**Published:** 2018-12-21

**Authors:** Eva Hilland, Nils I. Landrø, Catherine J. Harmer, Luigi A. Maglanoc, Rune Jonassen

**Affiliations:** ^1^Clinical Neuro-science Research Group, Department of Psychology, University of Oslo, Oslo, Norway; ^2^Division of Psychiatry, Diakonhjemmet Hospital, Oslo, Norway; ^3^Psychopharmacology and Emotional Research Laboratory (PERL), Department of Psychiatry, University of Oxford, Oxford, United Kingdom; ^4^NORMENT: Norwegian Centre for Mental Disorders Research, KG Jebsen Centre for Psychosis Research, Division of Mental Health and Addiction, Oslo University Hospital and Institute of Clinical Medicine, University of Oslo, Oslo, Norway; ^5^Faculty of Health Sciences, OsloMet—Oslo Metropolitan University, Oslo, Norway

**Keywords:** independent component analysis (ICA), salience network (SN), central executive network (CEN), default mode network (DMN), attention bias modification training (ABMT), major depression (MDD), resting state functional connectivity (RSFC)

## Abstract

Alterations in resting state networks (RSNs) are associated with emotional- and attentional control difficulties in depressed individuals. Attentional bias modification (ABM) training may lead to more adaptive emotional processing in depression, but little is known about the neural underpinnings associated with ABM. In the current study a sample of 134 previously depressed individuals were randomized into 14 days of computerized ABM- or a closely matched placebo training regime followed by a resting state magnetic resonance imaging (MRI) scan. Using independent component analysis (ICA) we examined within-network connectivity in three major RSN’s, the default mode network (DMN), the salience network (SN) and the central executive network (CEN) after 2 weeks of ABM training. We found a significant difference between the training groups within the SN, but no difference within the DMN or CEN. Moreover, a significant symptom improvement was observed in the ABM group after training.

**Clinical Trial Registration**: www.ClinicalTrials.gov, identifier NCT02931487.

## Introduction

Cognitive models of depression suggests that negatively biased attention is causally related to initiate and maintain depressive symptoms (Beck, 2008; Disner et al., 2011). Modifying biased attention has shown to produce change in depressive symptoms and implies a causal role for negative biases in depression (MacLeod et al., 2002; Wells and Beevers, 2010). Thus, it has been suggested that systematic modification of such biases may be integrated in the treatment and prevention of depression (Browning et al., 2012; Yang et al., 2015).

The neural underpinnings of attentional bias modification (ABM) training in depressed samples remains largely unknown. However, in a task-based functional magnetic resonance imaging (fMRI) study Browning et al. (2010b) reported that single session ABM was associated with lateral prefrontal cortex (PFC) reactivity towards emotional stimuli in healthy individuals, indicating moderation of neurocircuitry involved in emotion processing after ABM. In a recent study by our group we used a classical emotion regulation fMRI experiment, and found that ABM over 2 weeks was associated with reduced amygdala and anterior cingulate cortex (ACC) activation for negative images (Hilland et al., 2018). Moreover, we reported an improvement in depression symptoms after ABM. To this date there are only two studies on ABM in depressed populations investigating resting state functional connectivity (RSFC). Li et al. (2016) studied females with sub-threshold depression and found differences in spontaneous fluctuations between the right anterior insula and right middle frontal gyrus, areas known to play a key role in emotion processing. Beevers et al. (2015) found a group difference in connectivity within the middle frontal gyrus and dorsal ACC, a neural system important for emotion regulation. Difficulties with directing attention away from negative stimuli is thought to underlie biased attention in depression (Disner et al., 2011). Abnormalities in the three core intrinsic resting state network’s (RSN’s) the default mode- (DMN), central executive- (CEN) and salience networks (SNs) are implicated in emotional- and attentional control difficulties in depressed individuals (Gohier et al., 2009; Menon, 2011; De Lissnyder et al., 2012). Aberrant RSFC is also found in individuals with subthreshold depression compared to healthy controls and in individuals with high familial risk of depression (Ma et al., 2013; Hwang et al., 2016; Posner et al., 2016). The DMN, with its main nodes in posterior cingulate cortex (PCC) and ventromedial PFC (VMPFC), is thought to support internally oriented and self-referential information processing (Sheline et al., 2009). The CEN, comprising dorsolateral prefrontal- and inferior parietal regions is associated with focused attention on the external environment during demanding cognitive tasks (D’Esposito, 2007; Bressler and Menon, 2010). The SN, anchored in the dorsal ACC and bilateral anterior insula, is active when involved in processing emotional information and is important for assessing the relevance of internal and external stimuli in order to guide behavior (Greicius et al., 2007; Manoliu et al., 2014). Meta-analytic syntheses have shown that depression is characterized by decreased connectivity within the CEN, which is hypothesized to reflect decreased cognitive control of attention and emotion regulation. Moreover, depression has been associated with increased connectivity within the DMN, and between regions of the DMN and regions of the CEN. Depressed populations are also known to exhibit decreased connectivity between the SN and midline cortical regions that may mediate top-down regulation of emotions (Hamilton et al., 2012; Wang et al., 2012; Kaiser et al., 2015).

In the current randomized controlled trial (RCT) named Restoring Emotion Regulation Networks in Depression Vulnerability (NCT02931487) we used a sample of 134 participants previously treated for depression with various degrees of residual symptoms. Participants were scanned with a resting state MRI protocol shortly after a training period of 14 days of ABM or a closely matched placebo training condition (see Hilland et al., 2018). The study is, to the best of our knowledge, the first large scale study investigating RSFC on a depression group after ABM training. In the current clinical trial, we hypothesized that ABM would reshape dysregulated patterns of brain activity in neural circuits associated with depression. We presumed that ABM would affect neural systems supporting explicit regulation of emotion including cognitive control regions within the lateral- and PFC, which in turn should down-regulate core emotion processing limbic regions *via* midline structures of the ventral-rostral ACC. According to the preregistered hypothesis we anticipated increased integrity within attentional networks at rest as measured by independent component analysis (ICA) in ABM compared to neutral ABM training. Knowing that task-negative and task-positive RSN’s involved cognitive control of attention and emotion are dysregulated in depressed populations, we chose to analyze RSFC in three core RSN’s; the DMN, the SN and the CEN.

## Materials and Methods

### Participants and Screening Procedures

**Figure 1:**
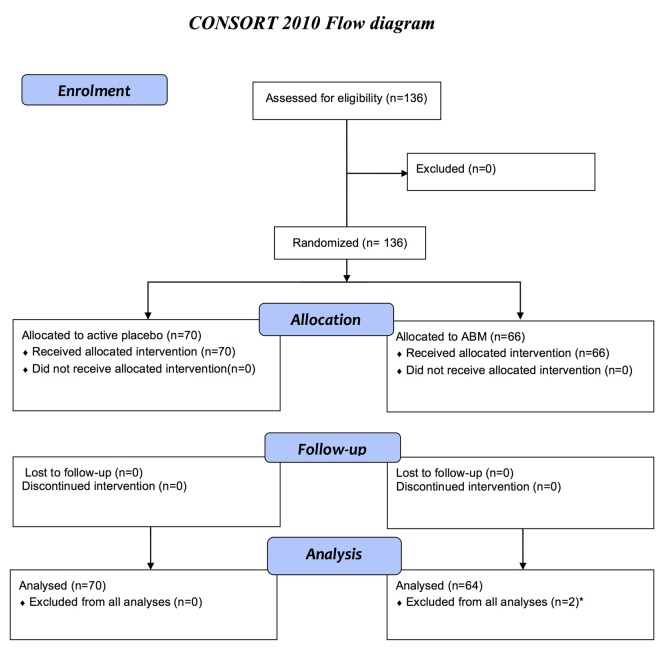
Flow diagram for enrolment, allocation to active placebo or attentional bias modification (ABM), follow-up after 2 weeks, and analyses in accordance with consort. The current clinical trial (NCT02931487) is an extension of a larger double-blinded randomized clinical trial (RCT; NCT02658682) including 321 patients with a history of depression. The current study is based on a subsample from the main RCT. Only participants that fulfilled the inclusion criteria per protocol was invited to participate in the magnetic resonance imaging (MRI) study, from May 2015 to December, 2016. The sample consists of 136 participants that agreed to participate in the MRI study and had no contraindication for MRI scanning. A total of 134 eligible participants between 18 years and 65 years old were included in MRI analyses. *Excluded due to technical problems with the head coil.

A total of 136 participants previously treated for MDD between 18 years and 65 years old were recruited from the main ABM RCT to the MRI RCT and randomized into two treatment conditions with either a positive ABM- or an evenly matched active placebo training (Figure 1). Block randomization was performed at inclusion to ensure balanced group characteristics. The current clinical trial is an extension of a larger double blinded RCT (NCT02658682) with 321 patients with a history of depression. Participants from this study were invited to participate in the fMRI study immediately after ABM training, and preferably within 1 week after completion. Participants were recruited mainly from an outpatient clinic in the Department of Psychiatry, Diakonhjemmet Hospital in Oslo, as well as from other clinical sites and *via* social media. Inclusion criteria: individuals that had experienced more than one depressive episode fulfilling the MINI A1a (depressed mood) and/or A2a (loss of interest or pleasure) criteria, more than 5 positive items on A3 and filling the A5 criterion (DSM 296.30–296.36 Recurrent/ICD-10 F33.x). Exclusion criteria: current major depressive episode according to MINI, current- or former neurological disorder, psychosis, bipolar spectrum disorders, substance use disorders, attention deficit disorder, or head trauma were. Beck Depression Inventory-II (BDI-II; Beck et al., 1996) and Hamilton Rating Scale for Depression (HRSD; Hamilton, 1960) was administered for clinician rated- and subjective evaluations of symptom severity. There was no upper or lower threshold on HRSD for inclusion. This study was carried out in accordance with the recommendations of The Regional Ethical Committee for Medical and Health Research for Southern Norway. Written informed consent from all participants was obtained before enrolment in accordance with the Declaration of Helsinki. The study protocol was approved by The Regional Ethical Committee for Medical and Health Research for Southern Norway (2014/217/REK sør-øst D).

### Attentional Bias Modification Procedure

The ABM training task was a computerized visual dot-probe procedure developed by Browning et al. (2012). A fixation cross was initially displayed followed by two images (the stimuli) presented concurrently on the top and bottom of the computer screen. Following stimulus onset, a probe (one or two dots) immediately appeared on the same location as one of the image stimuli and remained on the screen until the participant responded. The types of stimuli were pictures of emotional faces of three valences; positive (happy), neutral, or negative (angry and fearful). A single session of the task involved 96 trials with equal numbers of the three stimulus pair types. In addition, equal numbers of trials were randomly presented for 500- or 1,000 ms before the probe was displayed. In each trial stimuli from two valences were displayed, in one of the following pairing types: positive-neutral, positive-negative, and negative-neutral. In the ABM condition, probes were located behind positive stimuli in 87% of the trials (valid trials), as opposed to 13% with probes located behind the more negative stimuli (invalid trials). Consequently, participants should implicitly learn to deploy their attention toward positive stimuli, and develop a more positive attentional bias when completing the task. The neutral ABM placebo condition was otherwise identical, except the location of the probe, which was located behind the positive (valid trials) stimuli in 50% of the trials. Participants completed two sessions (96 trials) of ABM daily during the course of 14 days (28 sessions in total) on identical notebook computers (14″ HP EliteBook 840, 1,600 × 900, 8 GB, Intel Core i5-4310U), which were set up and used exclusively for ABM-training. Attentional bias scores were calculated as the difference in reaction time between trials in which the probe replaced the relatively more negative- vs. positive face. Thus, a more positive number reflects an increased bias towards more positive stimuli.

### Analysis of Symptom and Attentional Bias Change

Change in depression symptoms measured by HRSD was analyzed in PASW 25.0 (IBM) using a repeated measures ANOVA with training condition (ABM vs. placebo) as a fixed factor. The dependent variable was the difference score between symptoms at baseline and at 2 weeks follow-up. Analysis of change in attentional bias from baseline to follow up was done with a univariate ANOVA with condition as a fixed factor and pre bias score as a covariate.

### MRI Scan Acquisition

Scanning was conducted on a 3T Philips Ingenia whole-body scanner, with a 32 channel Philips SENSE head coil (Philips Medical Systems). Functional images were obtained with a single-shot T2* weighted single-shot gradient echo EPI sequence with the following parameters: repetition time (TR)/echo time (TE)/flip angle (FA) = 2,500 ms/30 ms/80°; voxel size, 3.00 × 3.00 × 3.00 mm; 45 transverse slices, 200 volumes; scan time ≈8.5 min. A T1-weighted 3D turbo field echo (TFE) anatomical image with SENSE using the following parameters: acceleration factor = 2; TR/TE/FA: 3,000 ms/3.61 ms/8°; scan duration: 3 min 16 s, 1 mm isotropic voxels. Participants were scanned with eyes open.

### MRI Analyses

fMRI data processing was done with the FMRI Expert Analysis Tool (FEAT) from the FMRIB Software Library (FSL; Smith et al., 2004). The procedure included brain extraction (BET), motion correction (MCFLIRT; Jenkinson et al., 2002), spatial smoothing (Gaussian kernel, full-width at half-maximum = 6 mm), high pass filtering (100 s) and single-session ICA (MELODIC). FSL’s MCFLIRT was used to compute estimated mean relative in-scanner head motion (volume-to-volume displacement). In order to automatically classify noise components and regress them out from the main signal FMRIB’S ICA-based Xnoiseifier (FIX) was used, with a threshold of 60 (Griffanti et al., 2014; Salimi-Khorshidi et al., 2014). In line with previous studies (Skåtun et al., 2016; Kaufmann et al., 2017), denoising substantially increased signal-to-noise ratio (*F*_(1,133)_ = 598.34, η^2^ = 0.81, *p* = 0.000) and none of the scans were deemed to have insufficient quality after denoising. T1-weighted structural images were skull-stripped using FreeSurfer 5.3 (Fischl et al., 2002), and used for registration to standard space (MNI-152) with FLIRT and boundary-based registration (BBR; Greve and Fischl, 2009) and FNIRT.

### Group ICA on fMRI Data

Analyses were performed using ICA with FSL-MELODIC and dual-regression. To allow the software to estimate the optimal number of components for each scan, model order was set to 0 which produced 21 spatial maps. IC spatial maps and corresponding time-series were estimated using dual the regression approach (Filippini et al., 2009). We assessed the spatial maps as well as the frequency profiles according to previous recommendations (Kelly et al., 2010). The CEN was split into two networks, a right- (RCEN) and a left lateralized component (LCEN). The DMN revealed a network comprising in the PCC, precuneus and medial PFC (MPFC). The SN revealed one network anchored in the ACC (SN) and temporal areas including the insula (see Supplementary Figure S1 for components of interest from group ICA).

Group analyses were performed in order to investigate difference in RSFC between ABM and placebo after training. Baseline symptom scores (Hamilton Depression Scale) and direction of MRI scan were used as covariates. FSL Randomise was run with 5,000 permutations. The clusters from the difference maps were determined using threshold-free cluster enhancement (TFCE), corrected for multiple comparisons across voxels for networks of interest (Winkler et al., 2014). Between group differences were considered statistically significant at *P* < 0.05.

## Results

### Baseline Symptoms

We found a significant difference in baseline symptoms between the two groups (Table 1). To account for this HRSD scores at baseline were added as a covariate in the design matrix used in FSL Randomise.

**Table 1:** Sample characteristics.

	Placebo (*n* = 70)	ABM (*n* = 64)	Value	Sig.
*Sample characteristics*				
Age	39.37 (13.55)	39.09 (12.80)	0.02	0.90
Gender (females)	44	47	1.71	0.02
Education level (ISCED)	5.89 (1.17)	5.85 (1.27)	0.06	0.84
Medication (SSRI)	23	22	0.03	0.86
Number of previous MDE	4.60 (5.33)	4.79 (7.56)	0.03	0.86
Days between ABM and fMRI	6.98 (8.43)	6.66 (6.96)	0.06	0.80
HRSD at baseline	7.53 (4.69)	9.56 (6.38)	4.22	0.04

*This table shows means, standard deviations together with *F*-values and Chi-square tests respectively. MDE, Major Depressive Episodes according to MINI; SSRI, any current use of an antidepressant belonging to the Selective Serotonin Reuptake Inhibitors; ISCED, International Standard Classification of Education*.

### Symptom Severity and Symptom Change After ABM

A statistically significant effect was found for change in depression symptoms measured by HRSD before and after intervention, with lower symptoms of depression in the ABM group (*F*_(1,133)_ = 4.277, η^2^ = 0.03, *p* = 0.041). Means and standard deviations at baseline in ABM were [9.56 (6.38)] and placebo [7.53 (4.69)], and changed to [7.94 (5.90)] and [7.77 (5.76)] at 2 weeks follow-up.

### Attentional Bias Change After ABM

Baseline corrected bias scores were 4.09 (2.25) in the ABM and 1.83 (2.18) in the placebo group, but this difference was not statistically significant (*F*_(1,133)_ = 0.515, *p* = 0.474).

### Effects of ABM on RSFC

**Figure 2:**
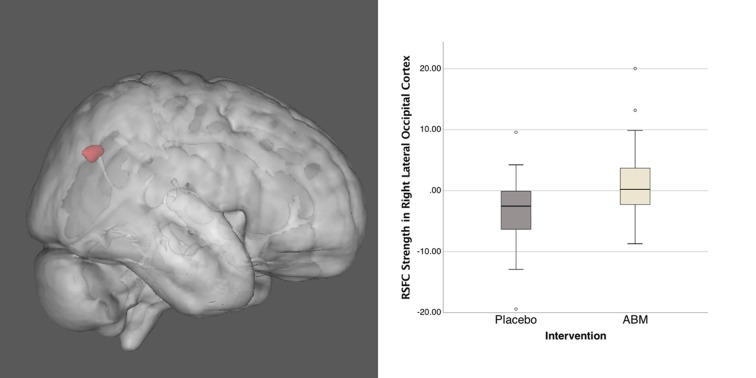
This image shows the group difference within the salience network (SN) for ABM over placebo (left), together with distribution of connectivity strength Z scores within this cluster (right). Activation height is thresholded at the *P* < 0.05 (corrected) level.

As part of our dual-regression probabilistic ICA approach, we identified 21 ICs in the temporally concatenated 4D population data set from which five ICs representing the DMN, CEN, and SN were robustly defined. Only one of the ICs of interest, the SN component, revealed significant group difference between ABM and placebo training group. For the SN the ABM > Placebo contrast revealed a cluster in the right lateral occipital cortex (MNI *x y z*; 32 −64 31; Figure 2). The cluster was located in an area comprising the angular gyrus (AG; BA39) and the superior parietal lobule (BA7).

## Discussion

The main finding of this study was higher RSFC in the AG/superior parietal lobule within the SN component in the ABM group compared to placebo after 14 days of training. No group differences were observed in the opposite contrast or within any other IC’s. Moreover, ABM training was associated with symptom improvement, compared to the placebo training condition, as previously reported (Hilland et al., 2018).

Altered connectivity in core RSN’s is thought to underlie cognitive and affective abnormalities in depression, yet little is known about the effects of ABM on these neural networks. The observed group difference within the SN was located in the right lateral occipital cortex, comprising the AG and the superior parietal lobule. The lateral occipital cortex is associated with visual object processing (Corbetta and Shulman, 2002; Bona et al., 2015), and has also been linked to facial perception (Grill-Spector et al., 2004). Large-scale connectivity analyses have indicated that the AG is one of the major connecting hubs, where converging multisensory information is combined and integrated in order to comprehend, manipulate mental representations, solve problems, and reorient attention to relevant information (Hagmann et al., 2008; Tomasi and Volkow, 2011; Seghier, 2013). Since ABM training procedures aim to shift attention away from negative- and towards more positive stimuli, the observed difference between training conditions could be interpreted as a sign of more adaptive attention and visual perception in the ABM group after training. Although the lateral occipital cortex is not part of the classical regions comprising the SN, group difference observed outside the boundaries of a specific RSN is not unusual in ICA analyses and such results demonstrate that connectivity between the specific area and the main regions of the SN is different in the two groups, despite on average not being strongly connected (see https://fsl.fmrib.ox.ac.uk/fsl/fslwiki/DualRegression/Faq).

Abnormal connectivity within regions of the SN has repeatedly been reported in depression samples (Horn et al., 2010; Lui et al., 2011; van Tol et al., 2013; Avery et al., 2014; Manoliu et al., 2014; Pannekoek et al., 2014), and new theories link dysfunction of the SN is to central depressive traits, including negatively biased information processing in MDD (Menon, 2011; Hamilton et al., 2016). The SN with its main nodes in bilateral anterior insula and ACC and major projections to subcortical- and frontal regions, is suggested to serve a general purpose in detecting salient information and initiate resources for appropriate behavioral responses (Seeley et al., 2007). The same network is also proposed to play a crucial role in switching between the DMN and the CEN (Menon and Uddin, 2010). Recent reviews conclude that depression is characterized by increased connectivity between the SN and the DMN (Mulders et al., 2015), and emphasize dysregulation in mood regulating circuits and the interaction between task-positive and task-negative networks (Wang et al., 2012). A meta-analysis by Kaiser et al. (2015) showed that depressed populations display hypoconnectivity between the SN and midline cortical regions, which may mediate top-down regulation of emotions. The meta-analysis by Hamilton et al. (2012) introduced a model describing how low responses of the DLPFC and striatum along with hyperreactivity of the SN towards negative stimuli, contributes to the pathophysiology of depression.

No differences within the DMN or the CEN following ABM were observed in the current study. The limited neuroimaging literature on ABM in depressed samples makes it challenging to interpret and evaluate our findings in a context. However, two studies examined the effects of ABM training in depression measured by RSFC, and both reported neural effects in brain areas implicated in detecting and responding to emotional information (Beevers et al., 2015; Li et al., 2016). Due to differences in experimental designs, patient groups, training procedures and RSFC analytic methods (e.g., seed-based vs. low frequency fluctuations vs. ICA) one cannot make direct comparisons to the results of the current study. Nevertheless, the neural systems described in the two studies included the insula and dACC, brain areas that overlap with central nodes of the SN.

In the present study symptom improvement was associated with ABM when compared to the placebo training group. Notably, there was an observed unbalance between the groups in symptom degree at baseline which may affect outcome variables. The ABM group had higher depression scores at baseline, and the symptom improvement could possibly reflect a regression toward the mean as these participants have more potential for symptom improvement. This likelihood could not be ruled out in the current study. To account for the baseline imbalance, symptom degree was added as a covariate in the group analysis, which should regress out variance related to this particular variable. However, conclusions and interpretations should be done with caution. The participants underwent scanning after training, but assessment at one time point does not allow statistical modeling of within-individual variance from baseline to follow-up, and may not allow causal relationships regarding changes in RSFC after ABM. Therefore, future studies should obtain MRI scans before and after ABM. The current sample is a group of patients with previous depression and different degrees of residual symptoms. Despite the study’s relatively large sample size, replicability should be tested in other patient groups. In the current study FIX was used for denoising, which significantly improved signal-to-noise ratio. Some studies suggest that the most effective approach to address artifacts in ICA is a combination of FIX and global signal reduction, e.g., Burgess et al. (2016). Future studies should include methods for reducing the global signal in the preprocessing stage.

In this study we used ICA, a model-free, data driven, exploratory technique which allows identification of resting-state networks and noise components from spontaneous brain activity. One main advantage of the ICA approach is that it allows you to separate resting fluctuations from other structured noise-related signal variations (Damoiseaux et al., 2006).

ICA methods are designed to search for a mixture of underlying sources that can explain the resting-state patterns, looking for the existence of spatial sources of signals that are maximally independent from each other (van den Heuvel and Hulshoff Pol, 2010). As opposed to the seed-based approaches, ICA is objective in that the results cannot be biased by *a priori* regions of interest. A possible detriment is of ICA is that the components are based on highly complex representations of neural data, which could complicate the translation of between-group results to clinical relevance (Fox and Raichle, 2007). ICA is underrepresented in the depression literature compared to hypotheses-driven analytic methods (see e.g., Kaiser et al., 2015). Seed-driven approaches are the most widely used, and typically describe the influence one brain area exerts over another area. Seed-based methods can establish connectivity between any two synchronous voxels independent of their allegiance to intrinsically connected networks (Joel et al., 2011). Such analytic approaches are advantageous in that they allow confirmation of research questions closely linked to psychological theory and phenomena within predefined brain areas (Cole et al., 2010), and interpretations of the results may in general be more straight forward. A limitation to seed-based analysis is that activation of one functional network cannot be distinguished from co-activation of other networks, which leads to the same limitation as task-fMRI studies using the general linear model, the problem of “cognitive subtraction”(Friston et al., 1996). Large scale RSN’s of the human brain measured by both ICA/dual regression as well as seed-based methods have shown moderate to high test-retest replicability (Shehzad et al., 2009; Biswal et al., 2010; Zuo et al., 2010). While there are distinct advantages and disadvantages to the different methods, the lack of ICA studies has been highlighted as an opportunity for researchers to provide further insights to the depression field (Wang et al., 2012).

ABM procedures were initially developed to study associations between attentional biases and variation in emotional vulnerability in experimental settings (MacLeod et al., 2002). Over the past decade the interest in research on cognitive biases have accelerated, particularly clinical studies aiming to find therapeutic benefits for anxiety and depressive disorders (Grafton et al., 2017). Some studies and meta-analyses have shown mixed findings (see Peckham et al., 2010; Cristea et al., 2015), while other studies have given promising results indicating that ABM reduces symptoms and may prevent relapse of new depressive episodes (Browning et al., 2010a; Linetzky et al., 2015; Yang et al., 2015). The results of the current study indicate an effect of ABM on depression symptoms, along with increased connectivity within the SN. To disentangle the effects of ABM, future studies should also investigate the neural basis of ABM training with other emotional valence categories, e.g., mood congruent (sad) facial stimuli.

## Conclusion

This study revealed increased connectivity within the SN in brain areas linked to perception, attention and emotion processing after ABM, as well as a significant improvement in depression symptoms, which supports previous findings that ABM modifies both symptoms and brain function in psychopathology. The translation of experimental psychopathology and neuroimaging research to reduce depressive symptoms may help delineate the mechanisms underlying development and recurrence of depressive episodes.

## Data Availability

The datasets generated for this study are available on request to the corresponding author.

## Author Contributions

EH and RJ was involved in the conception and design, data acquisition, data analysis and interpretation of data, drafting and reviewing the article and gave final approval to the version. NL and CH contributed in the conception and design, interpretation of data, reviewed the article and approved the version. LM was involved in the data acquisition, statistical analysis, reviewing the article and gave final approval of the version.

## Conflict of Interest Statement

CH has received consultancy fees from Johnson and Johnson Inc, P1 vital and Lundbeck. NL has received consultancy fees and travel expenses from Lundbeck. The remaining authors declare that the research was conducted in the absence of any commercial or financial relationships that could be construed as a potential conflict of interest.
