# Supplementary figures and images for: Within-Network Connectivity in the Salience Network After Attention Bias Modification Training in Residual Depression: Report From a Preregistered Clinical Trial
Source: Front Hum Neurosci. 2018 Dec 21;12:508. doi: 10.3389/fnhum.2018.00508 (PMC6308203; doi:10.3389/fnhum.2018.00508)

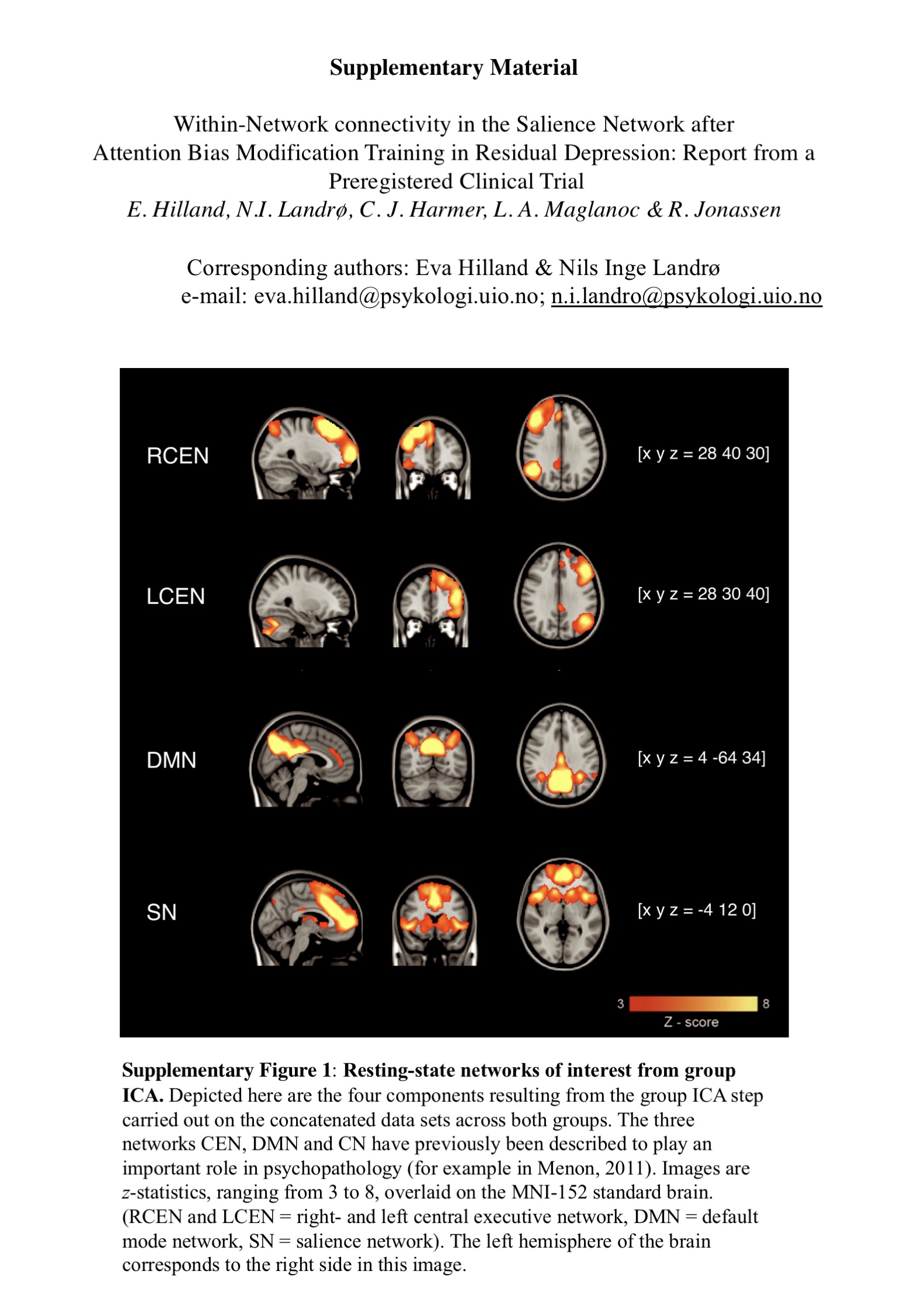

Supplement: Supplementary file 1 [file Image_1.JPEG]
